# ARF degradation defines a deeply conserved step in auxin response

**DOI:** 10.1038/s41477-025-01975-1

**Published:** 2025-04-11

**Authors:** Martijn de Roij, Jorge Hernández García, Shubhajit Das, Jan Willem Borst, Dolf Weijers

**Affiliations:** 1 Laboratory of Biochemistry, Wageningen University, Wageningen, the Netherlands; 2 Present Address: Institute for Science and Technology Austria, Klosterneuburg, Austria

**Keywords:** Auxin, Plant development, Phylogenetics

## Abstract

In land plants, the signalling molecule auxin profoundly controls growth and development, chiefly through a transcriptional response system. The auxin response is mediated by modulating the activity of DNA-binding auxin response factor (ARF) proteins. The concentrations and stoichiometry of the competing A- and B-class ARFs define cells’ capacity for auxin response. In the minimal auxin response system of the liverwort *Marchantia polymorpha*, both A- and B-ARFs are unstable, but the underlying mechanisms, developmental relevance and evolutionary history of this instability are unknown. Here we identify a minimal motif that is necessary for MpARF2 (B-class) degradation and show that it is critical for development and the auxin response. Through comparative analysis and motif swaps among all ARF classes in extant algae and land plants, we infer that the emergence of ARF instability probably occurred in the ancestor of the A- and B-ARF clades and, therefore, preceded or coincided with the origin of the auxin response system.

## Abstract (web summary)

De Roij et al. map a minimal region for auxin response factor (ARF) degradation in *Marchantia* and reveal that ARF instability predates the emergence of the auxin response. This identifies ARF degradation as a key and ancestral property of the auxin response.

## Main

Auxin is a central signalling molecule in plant development^1^ and acts chiefly through the activation of auxin response factors (ARFs), a family of DNA-binding transcription factors^2,3^. In land plants, ARFs are phylogenetically divided into three classes (A, B and C)^4^. A-ARFs are auxin-dependent gene regulators and are antagonized by auxin-independent B-ARFs^5,6^. Based on work in the liverwort *Marchantia polymorpha*, C-ARFs appear detached from the auxin response^5^. Given that competition between A- and B-ARFs determines the auxin response, their levels and stoichiometry are key parameters defining this response. We recently developed fluorescent knock-in reporters of all auxin response proteins in *Marchantia*^7^, which encodes single copies of each ARF class. We found that both A-class (MpARF1) and B-class (MpARF2) ARF proteins are unstable and that this instability requires the 26S proteasome^7^. Several other ARFs have been reported to be targeted for degradation (reviewed in ref. ^8^), but many questions regarding the mechanisms, biological importance and evolutionary origin of the instability remain.

**Fig. 1:**
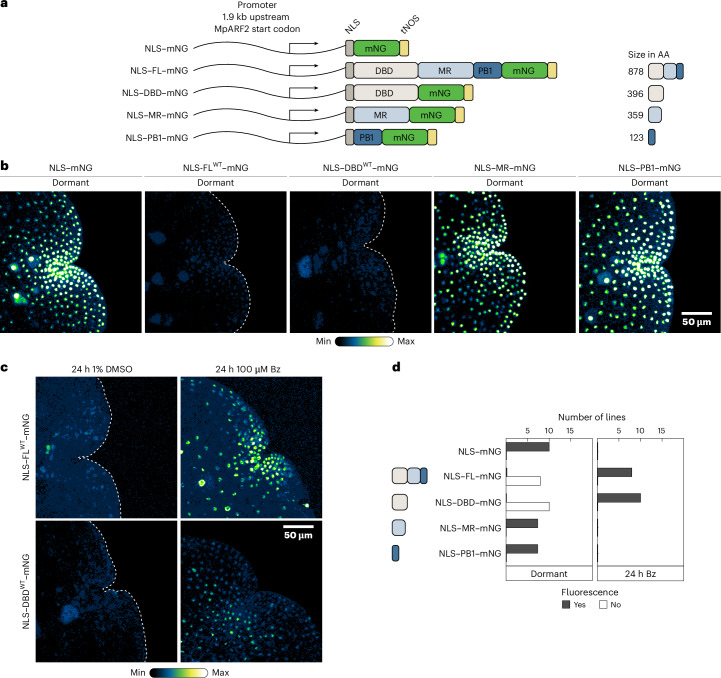
The DNA-binding domain confers MpARF2 instability. **a**, A schematic overview of translational fusion proteins assessed in subsequent assays. AA, amino acids. **b**, Confocal images of *M. polymorpha* gemmae, showing the expression of fusion proteins outlined in **a**. **c**, Gemmae were treated with Bz or DMSO and imaged by confocal microscopy. Proteins correspond to **a**. **d**, Quantification of fluorescence patterns shown in **b** and **c** in a set of independent transgenic lines. If no fluorescence in dormant gemmea was detected (left), plants were treated with Bz and fluorescence was reassessed (right).

**Extended Data Fig. 1:**
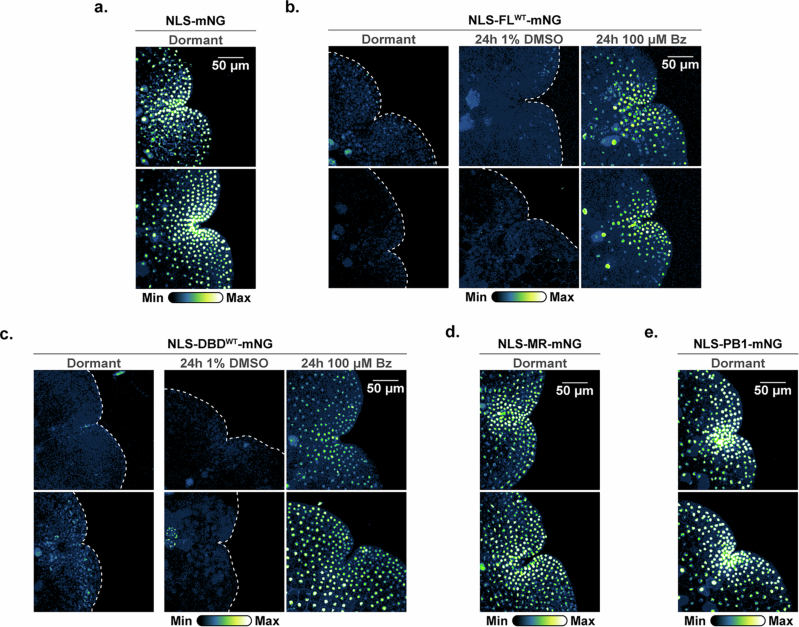
Assessment of stability of fusion proteins through confocal microscopy. **a**–**e**. Confocal images of *M. polymorpha* gemmae, showing representative expression of different fusion proteins as outlined in Fig. 1d. Here we show protein behavior in two additional independent lines for all panels in Fig. 1. Bz, Bortezomib.

**Extended Data Fig. 2:**
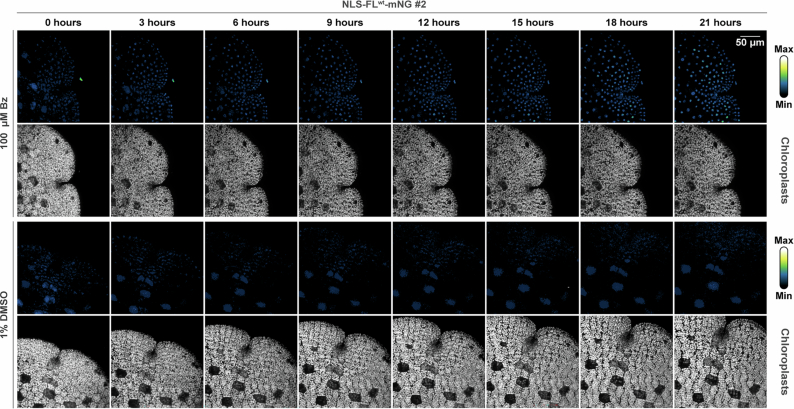
Dynamics and proteasome-dependence of NLS-FL^wt^-mNG accumulation. Time series of gemma expressing NLS-FL^wt^-mNG treated with Bortezomib (Bz) or DMSO for 21 h.

To address these questions, we first mapped the region in MpARF2 conferring instability. We expressed mNeonGreen (mNG) fluorescently tagged, nuclear-targeted protein domains from the native *MpARF2* promoter (Fig. 1a). While the nuclear localization signal (NLS) control (NLS–mNG) accumulated to high levels, Full-Length (FL) MpARF2 (NLS–FL–mNG) could not be detected (Fig. 1a,b,d) unless the 26S proteasome was inhibited with bortezomib (Bz; Fig. 1c and Extended Data Figs. 1a,b and 2), as expected. While mNG fusions to the middle region (MR; NLS–MR–mNG) or Phox and Bem1 domains (PB1; NLS–PB1–mNG) were stable (Fig. 1a,b,d), the DNA-binding domain (DBD; NLS–DBD–mNG) alone was sufficient to confer instability (Fig. 1a,b,d and Extended Data Fig. 1c–e). Again, Bz treatment led to protein accumulation (Fig. 1c,d and Extended Data Fig. 1c). Thus, the MpARF2 DBD contains the minimal elements required for instability.

**Fig. 2:**
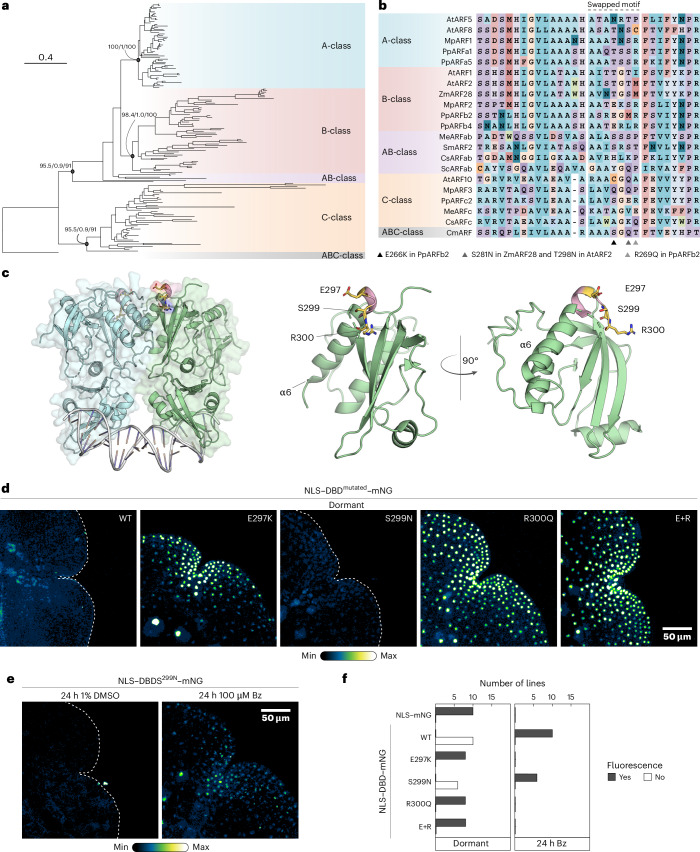
Identification of a motif in MpARF2 essential for instability. **a**, Phylogenetic relationships between ARFs. Statistical support for major nodes is shown as SH-aLRT (Shimodaira–Hasegawa approximate likelihood ratio test) branch test/approximate Bayesian test/ultrafast bootstrap approximation values. **b**, Multiple sequence alignment of ARFs representing the major classes. Putative amino acids responsible for instability (and the swapped motifs) are outlined. **c**, The MpARF2 DBD crystal structure with key residues E297, S299 and R300 highlighted and enlarged. **d**, Confocal images of gemmae expressing NLS–DBD–mNG fusions carying outlined mutations. **e**, Gemmae were treated with Bz or DMSO and imaged by confocal microscopy. **f**, Similar to Fig. 1d, the same controls (NLS–mNG and NLS–WT–mNG) were plotted, but now compared to NLS–WT–mNG carrying the indicated mutations.

**Extended Data Fig. 3:**
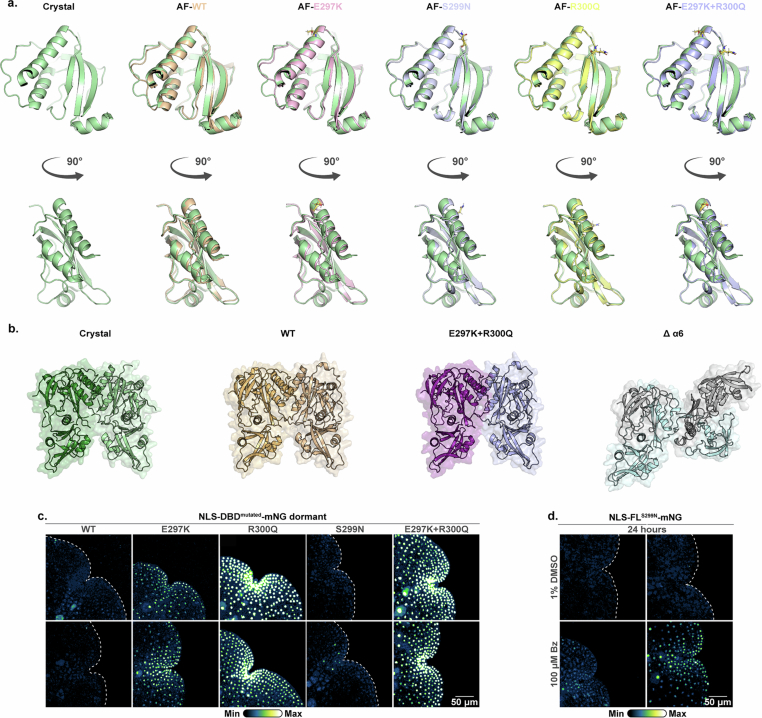
AlphaFold2 models of mutated residues and dimerization capacity. **a**. Overview of the introduced point mutations within the MpARF2 DBD. Mutations were visualized through AlphaFold2 modelling and projected onto the experimentally verified (X-ray crystallography) MpARF2 DBD shown in green. **b**. AlphaFold2 models of homodimerization of the WT MpARF2 DBD, the E297K+R300Q mutant, and a version where α-helix 6 was deleted (right) when compared to the structure of MpARF2 (left, green). **c**, **d**. Confocal images of *M. polymorpha* gemmae, showing representative expression of different fusion proteins as outlined in Fig. 2d, e. Here we show protein behavior in two additional independent lines for all panels in Fig. 2. Bz, Bortezomib.

Recently, mutations in the DBD of *Physcomitrium patens* and *Zea mays* (maize) B-ARFs were shown to inhibit proteasome-dependent degradation. In both cases, mutations map to the same short motif and engineering an equivalent mutation in *Arabidopsis thaliana* AtARF2 also reduced breakdown^9^. It is therefore possible that instability in B-ARFs has a single, common origin in ARF evolution (Fig. 2a). The existence of a single B-ARF in *Marchantia* enables the testing of this hypothesis. Indeed, the amino acids required for instability in *Physcomitrium* and maize are conserved in MpARF2 (E297, S299 and R300 in MpARF2; Fig. 2b). When mapped onto our experimentally determined MpARF2 DBD structure^5^, we found these to be positioned in an outward-facing loop towards the C-terminus of α-helix 6, close to the DBD dimerization interface (Fig. 2b,c). Despite the proximity of the mutations that interfere with degradation in *Physcomitrium*, maize and *Arabidopsis* B-class ARFs to this interface, AlphaFold-based structural models of of the mutations in MpARF2 (Extended Data Fig. 3a,b), suggest that homodimerization may not be affected.

Next we engineered E297K, S299N and R300Q single mutations, as well as E297K+R300Q (E+R) double mutations in the NLS–DBD–mNG protein and analysed accumulation and stability (Extended Data Fig. 3a). All mutations except S299N led to strong nuclear signals (Fig. 2d–f and Extended Data Fig. 3c,d). By contrast, the S299N mutation did not stabilize the protein (Fig. 2d–f and Extended Data Fig. 3c,d). Thus, B-ARF degradation probably has a single evolutionary origin, but, as discussed in ref. ^9^, there probably is co-evolution between the ARF and its proteolysis partner.

**Fig. 3:**
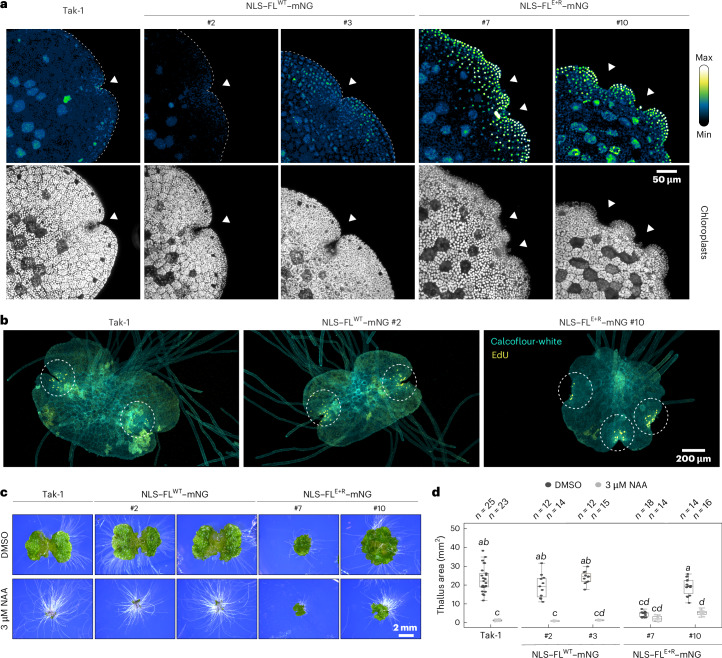
Biological importance of MpARF2 degradation. **a**, Confocal images of Tak-1 wild-type and transgenic gemmae expressing NLS–FL^WT^–mNG or NLS–FL^E+R^–mNG constructs. Chloroplast autofluorescence outlines gemmae morphology. The hashed numbers are independent transgenic lines. White arrowheads designate apical notches. **b**, Confocal images of EdU staining in 3-day-old gemmalings of S-phase nuclei (yellow). Cell walls were stained with calcofluor-white (turquoise). **c**, A qualitative comparison of the auxin response in gemmalings grown for 10 days on medium containing 3 μM NAA or DMSO, respectively. **d**, Quantification of projected thallus area of gemmalings shown in **c** compared per one-way ANOVA (*F* = (9, 152) = 122.6, *P* ≈ 2 × 10^−16^) with Tukey post-hoc test with Benjamini–Hochberg correction (italics denote significant differences, *P* < 0.05). The box plots show the following statistical parameters: central line, median; upper bound, first quartile; lower bound, third quartile; whiskers show the highest and lowest values, respectively, within 1.5× interquartile range. Individual gemmae were selected randomly and grown as independent biological replicates, the sample size is. Raw data and exact *P* values are provided as a Source dataset. Source data

**Extended Data Fig. 4:**
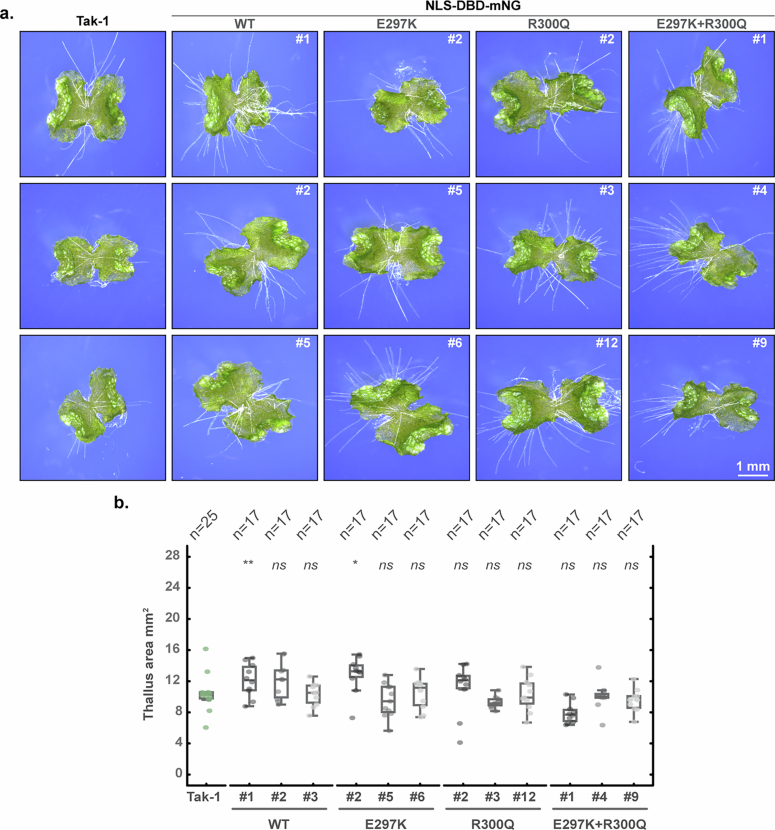
Expression of stabilized NLS-DBD-mNG fusions has no effect on Marchantia development. **a**. Overview of phenotypes of seven day old gemmalings grown on ½ strength Gamborgs B5 medium wich express the NLS-DBD-mNG fusion proteins which harbour no mutations (WT) or mutations in key residues for stability as shown in Fig. 2d. **b**. Projected thallus area of aforementioned plants were measured and statisticaly compared by one-way ANOVA (*F* = (14, 216) = 3.841 *p* ≈ 7.94e^−6^) with Tukey post-hoc test (asterixes denote significant differences from Tak-1, * *p* < .05; ** p < .01). Boxplots show the following statistical parameters: central line, median; upper bound, first quartile; lower bound, third quartile; whiskers show the highest and lowest values, respectively, within 1.5*inter-quartile range (IQR). Individual gemmae were selected randomly and grown as independent biological replicates. The sample size is indicated and represents individual gemmalings. Raw data and exact p-values are provided as a. Source data

**Extended Data Fig. 5:**
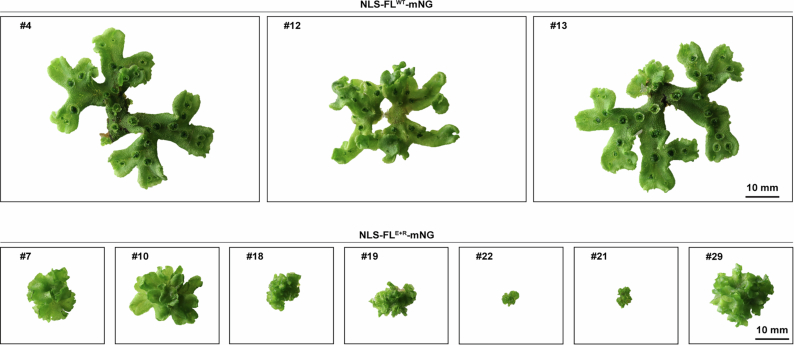
Expression of a stabilized full-length MpARF2 protein disturbs normal Marchantia development. Overview of representative phenotypes of plants which survived T_1_ selection on hygromycin-containing ½ Gamborgs B5 medium for 50 days. Compared here are multiple independent transgenic lines expressing NLS-FL^WT^-mNG (top panel) and NLS-FL^E+R^-mNG (bottom panel, harbours the E297K and R300Q mutations).

**Extended Data Fig. 6:**
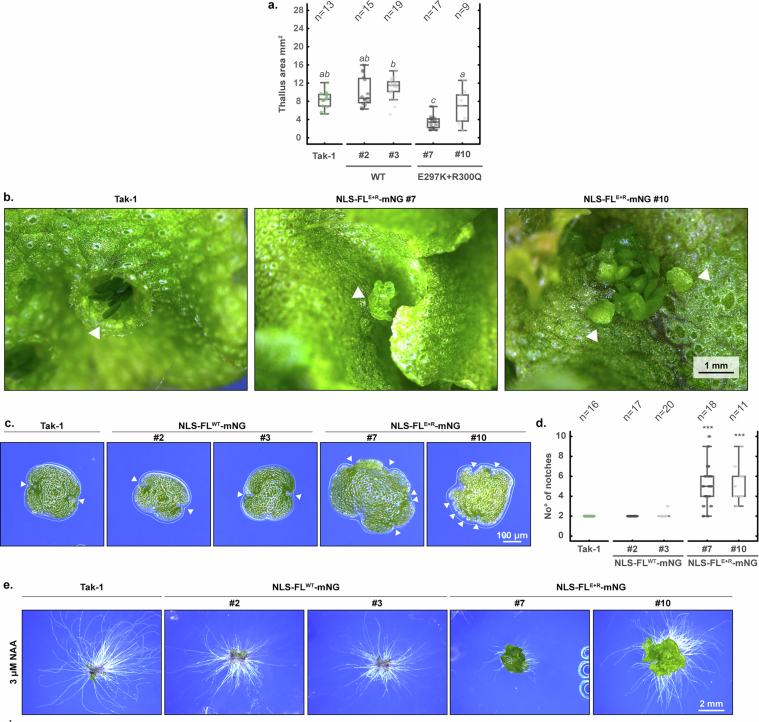
NLS-FL^E+R^-mNG mutants exhibit pleitropic developmental defects and impaired auxin response. **a**. Projected thallus area of seven day old gemmalings grown on ½ strength Gamborgs B5 medium. A comparison is made between the Tak-1 WT and plants wich express the NLS-FL^WT^-mNG and NLS-FL^E+R^-mNG fusion proteins (shown are two independent lines per construct). Thallus area was statisticaly compared by one-way ANOVA (*F* = (4, 68) = 24.3, *p* ≈ 1.65e^−12^) with Tukey post-hoc test (italics denote significant differences, *p* < .05). Boxplots show the following statistical parameters: central line, median; upper bound, first quartile; lower bound, third quartile; whiskers show the highest and lowest values, respectively, within 1.5*inter-quartile range (IQR). **b**. Detailed images of gemmae formation in gemma cups (Tak-1, left) and on the thallus (NLS-FL^E+R^-mNG #7 and #10, wherein hashes indicate independent transgenic lines). **c**. Stereomicroscope images of dormant gemmae of plants described in a. **d**. When clearly distinguishable apical notches of gemmae in c. were observed, these were counted and were statistically compared by a Chi-squared test (*** *p* < .001) to the Tak-1 WT. Boxplots show the following statistical parameters: central line, median; upper bound, first quartile; lower bound, third quartile; whiskers show the highest and lowest values, respectively, within 1.5*inter-quartile range (IQR). Individual gemmae were selected randomly and grown as independent biological replicates. The sample size is indicated and represents individual gemmae. **e**. Overview of gemmalings of plants described in a. were grown on 3 μM NAA containing ½ Gamborgs B5 medium for 17 days (same experiment as Fig. 3c but plants were grown for an additional seven days). For a. & d. the sample size is indicated and represents individual gemmalings, individual gemmae were selected randomly and grown as independent biological replicates. The source data and exact p-values are provided as a. Source data

We deliberately engineered mutations in the DBD, to uncouple degradation and accumulation from potential effects on plant growth and development (Extended Data Fig. 4a,b). It is, however, unclear what biological relevance MpARF2 degradation has. To address the impact of a lack of MpARF2 degradation on growth, development and auxin response, we engineered the E+R mutation in the full-length fusion protein. As expected, NLS–FL^E+R^–mNG-bearing gemmae exhibited very high levels of nuclear mNG fluorescence, whereas lines expressing NLS–FL^WT^–mNG (where WT means wild type) did not unless they were treated with Bz (Figs. 1b,c and 3a). MpARF2-accumulating lines displayed a wide range of strong developmental defects, none of which was observed in NLS–FL^WT^–mNG lines (Extended Data Fig. 5). Most NLS–FL^E+R^–mNG lines were dwarfed, showed epinastic growth and ectopic apical notch formation, and failed to produce gemmae (Extended Data Figs. 5 and 6a). Some lines did form gemmae (#7 and #10), even though they mostly did so on the thallus, rather than inside gemmae cups (Extended Data Fig. 6b). However, these lines allowed us to explore effects of MpARF2 accumulation on gemmae development and auxin response. Mature gemmae showed defects in apical (meristematic) notch formation, often showing many more notches than the regular two notches (Fig. 3a and Extended Data Fig. 6c,d). The 5-ethynyl-2′-deoxyuridine (EdU) staining of S-phase cells confirmed the existence of supernumerary notches (Fig. 3b). As expected from B-ARF accumulation, NLS–FL^E+R^–mNG gemmae showed a strong reduction in auxin response (Fig. 3c,d and Extended Data Fig. 6e). Thus, regulation of MpARF2 levels through proteasomal degradation is necessary for normal development and auxin response.

**Fig. 4:**
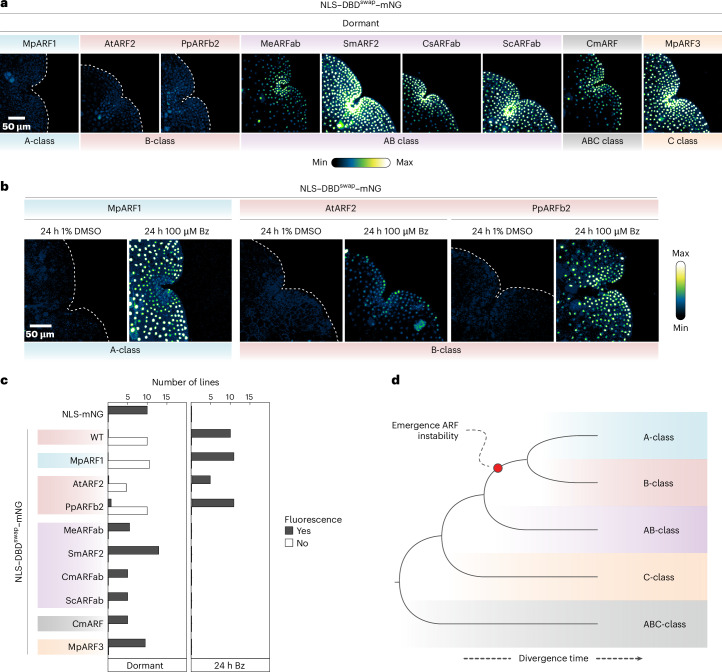
The evolutionary origin of ARF instability. **a**, Fluorescence accumulation (assessed by confocal microscopy) of swaps of homologous motifs of different ARFs (representing major ARF classes) into NLS–DBD–mNG. **b**, Lines that showed no detectable fluorescence in dormant gemmae in **a** were treated for 24 h with Bz or DMSO, and fluorescence accumulation was reassessed. **c**, Similar to Fig. 1d, the same controls (NLS–mNG and NLS–WT–mNG) were plotted, but now compared to NLS–WT–mNG carrying various motif swaps, as indicated. **d**, A schematic cladogram illustrating the hypothesized evolutionary relationship between known ARF clades and the emergence of the instability-conferring motif.

**Extended Data Fig. 7:**
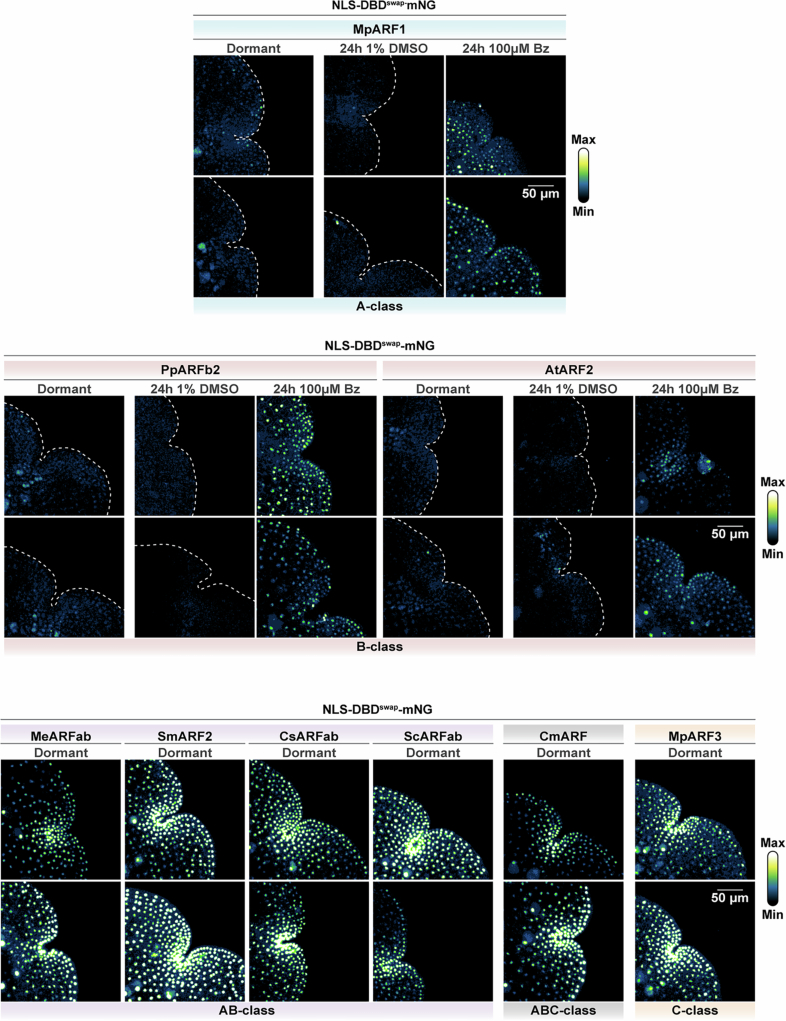
Investigation of the ability of homologous regions of ARF proteins from different Streptophyte species in conferring instability. Confocal images of *M. polymorpha* gemmae, showing expression of different NLS-DBD-mNG fusion proteins with homologous sequences of other ARFs swapped. Here we show protein behaviour in two additional independent lines for all panels in Fig. 4. Bz stands for Bortezomib.

Studies in several species show that equivalent mutations stabilize *Marchantia*, *Physcomitrium*, maize and *Arabidopsis* ARFs, but given that these are all studied in separate contexts, it cannot be concluded that these reflect the same mechanism. We therefore tested if these motifs are functionally equivalent by swapping the *Physcomitrium* PpARFb2 and *Arabidopsis* AtARF2 motifs into MpARF2 DBD. The NLS–DBD^PpARFb2-swap^–mNG and NLS–DBD^AtARF2-swap^–mNG proteins were unstable, yet accumulated when treated with Bz (Fig. 4a–c and Extended Data Fig. 7). Thus, the degradation mechanism is probably homologous between species.

Given that, in *Marchantia*, both A- and B-ARFs are unstable^7^, and having identified the minimal region that is both necessary and sufficient for MpARF2 degradation, we next asked if mechanisms of instability are universal among A- and B-ARFs. There is some conservation between MpARF1 and MpARF2 in the region required for MpARF2 (and *Physcomitrium*, maize and *Arabidopsis*) instability (Fig. 2b). We tested if the equivalent region from MpARF1 could render MpARF2 unstable, that is, we swapped this short motif into the MpARF2 NLS–DBD–mNG (Fig. 2b). As a control, we included the equivalent region from the C-class MpARF3, which is stable in its native context^7^. While the NLS–DBD^MpARF3-swap^–mNG protein accumulated to high levels even in the absence of Bz, we could hardly detect NLS–DBD^MpARF1-swap^–mNG protein (Fig. 4a–c and Extended Data Fig. 7). When treated with Bz, however, this protein accumulated to high levels (Fig. 4b,c and Extended Data Fig. 7). Thus, homologous motifs in MpARF1 and MpARF2 confer comparable protein instability. A- and B-ARFs derive from duplication in an ancestral AB-ARF gene, which, in turn, originates from a split of an ancestral ABC-ARF thereby forming the AB- and C-ARF clades^4,10^, and neofunctionalized following this duplication (Fig. 2a)^10^.

The finding that the same region in MpARF1 and MpARF2 can confer instability suggests that this feature has a single origin that predates the divergence of A- and B-ARFs. Although the nature of the ancestral ABC- and AB-ARFs is not clear, extant representatives of these proteins are found in the sister lineages to land plants, the streptophyte algae^4^. We selected representative AB-ARFs of algae belonging to the Zygnematophyceae group, the lineage most closely related to land plants^11^ (*Mesotaenium endlicherianum* MeARFab; *Spirogloea muscicola* SmARF2), as well as from Coleochaetophyceae (*Coleochaete scutata* CsARFab) and Klebsormidiophyceae (*Streptosarcina costaricana* ScARFab) clades. We also included a member of the Chlorokybophyceae (*Chlorokybus melkonianii* CmARF) representing the ABC-ARF clade^11^. We swapped the homologous motifs from these ARFs (Fig. 2b) into NLS–DBD–mNG. All of these swapped fusions were highly stable, mimicking the behaviour of fusion proteins with mutated degron residues (Fig. 4a,c and Extended Data Fig. 7). This result is consistent with two interpretations: one would be that the ancestral state of either all ARFs or AB-ARFs included protein instability conferred by this motif, followed by subsequent loss in the lineages giving rise to the tested algal ARFs, which is unlikely. Alternatively, the ancestral ARF state was stable, and instability evolved in the lineage giving rise to land plants before A/B divergence (Fig. 4d). The very long evolutionary divergence times in this group of algae (>600 Mya (ref. ^12^)) and sparse species sampling make such inferences problematic.

Our work identifies a minimal region for ARF degradation in *Marchantia* and shows that a key element required for instability is conserved among B-ARFs, even in *Marchantia*, which has only a single copy. We show that MpARF2 instability is critical for normal development and auxin response. Our analysis of MpARF1 reveals that the capacity to mediate instability is very likely to be an ancestral property of the protein that predated the split between A-ARFs and B-ARFs.

While the minimal motif from MpARF1 can destabilize MpARF2 when transplanted, an interesting question is whether the same minimal region is also required for the degradation of MpARF1 under natural conditions. MpARF1 is unstable, and proteasome inhibition prevents a decline in protein levels. This, however, differs from MpARF2, where proteasome inhibition not only prevents degradation but actually leads to protein hyperaccumulation^7^. While this may reflect different feedback controls triggered by either MpARF1 or MpARF2 accumulation, it may also reflect that mechanisms of degradation are not identical for the two proteins. Further analysis of sequence requirements for MpARF1 degradation should resolve this.

The minimal motif for ARF degradation is located within the DBD, close to the dimerization interface. An open question is if and how degradation and dimerization interact. Does degradation prevent the accumulation of monomers? Does dimerization protect ARFs from degradation? Or are these two completely separate properties? Given how closely the two regions are linked, it may not be trivial to separately interfere with the two.

Based on genetic analysis of the *Marchantia* auxin response system^5,10^, auxin dependence evolved only after the A/B split^10^. We therefore infer that ARF instability either co-emerged with or preceded the origin of the auxin response system. A key future question is by what mechanism the minimal region confers instability. This region probably constitutes a protein–protein interaction interface that allows a ubiquitin ligase, or another proteolysis adaptor protein, to bind. The fact that the S299N mutation does not stabilize MpARF2, while the orthologous mutation stabilizes maize ZmARF28, suggests divergence in the degradation interface, and likely co-evolution with a partner protein. We expect that future identification of other components in ARF degradation will help in understanding the mechanisms underlying ARF degradation, including an understanding of the sequence requirements in the minimal degradation motif. Identification of additional components will probably also help to explain the diversity and biological significance of ARF degradation across the many processes that auxin controls.

## Methods

### Sequence identification, alignment and phylogenetic analysis

Most ARF sequences have been previously identified^4^. Additional sequences have been retrieved through BLASTP analysis using reference databases for *Zea mays* (B73-NAM-5.0.55), *Ceratopteris richardii* (V2.1), *Selaginella kraussiana*, *Ceratodon purpureus* (GG1) and algal genomes of *Mesotaenium endlicherianum* (SAG12.97), *Chlorokybus melkonianii* (CCAC 0220, previously *C. atmophyticus*) and *Zygnema circumcarinatum*, all accessed through Phycocosm. For *M. endlicherianum*, the v2.0 annotation was surveyed to obtain the MeARFab sequence. The BLASTP cut-off was set to 10 × 10^−10^ for algal searches. All sequences were first checked for the presence of ARF DNA-binding domain features (that is, B3 DNA-binding domain, PF02362; and Auxin_resp, PF06507) or discarded otherwise. Protein sequences were aligned with the E-INS-i algorithm of MAFFT version 7 (https://mafft.cbrc.jp/alignment/software/). Alignments were trimmed to keep the DBD sequences and manually curated. TrimAI was used on curated alignments to discard positions with more than 80% gaps (http://trimal.cgenomics.org/). The ModelFinder implementation in IQ-TREE was used on final alignments to choose JTT+I+G4 as substitution model based on the Akaike and Bayesian information criteria. IQ-TREE was also used on the final alignment to build a maximum likelihood phylogenetic tree. Branch statistic support was inferred using ultrafast bootstrap (5,000 alignments), the SH-like approximate likelihood ratio test and approximate Bayes test. The resulting tree, rooted to the ABC-ARF CmARF, can be found at https://itol.embl.de/tree/13722425211476661730903969. The tree figure was manually adapted as a vectorial image keeping the scale parameters.

### Structural analysis degron

The MpARF2 crystal structure was retrieved from the Protein Data Bank (https://www.rcsb.org/structure/6SDG)^5^. Structural visualization was performed using Pymol (v2.3.4) software. To model the mutated versions of the MpARF2 DBD, we used AlphaFold2-multimer v2.2.2 (ref. ^13^).

### Plasmid construction

A genomic fragment 1,920 bp upstream of the *MpARF2* start codon was amplified with primers MdR298 and MdR299 (Supplementary Table 1). This region was used as a promoter to drive expression of the transgenes. Next, the plasmid pMpGWB100, carrying a hygromycin resistance casette, was digested with the restriction enzyme XbaI (Thermo Fisher Scientific), and the aforementioned *MpARF2* promoter was ligated downstream of an XbaI site followed by the mNG coding sequence using NEBuilder HiFi DNA Assembly (New England Biolabs)^14^. Next, MpARF2 domains, the full-length protein coding sequence and mutant versions thereof were amplified from in-house plasmids using primer pairs specified in Supplementary Table 1 and introduced into the XbaI site flanked by the *MpARF2* promoter and mNG. The degron swap constructs were amplified in two fragments from the MpARF2 DBD with two primer pairs containing a non-complementary sequence to introduce the swap. These fragments were then integrated using HiFi DNA Assembly (Supplementary Information Table 1). Swaps were generated on the basis of the alignment shown in Fig. 2b.

### Plant growth conditions and transformation

For this study, *Marchantia polymorpha* Takaragaike-1 (Tak-1) was used as the wild type. Plants were grown on ½ strength Gamborg B5 medium at 22 °C with 40 μmol photons m^−2^ s^−1^ of continuous white fluorescent light. Tak-1 was transformed using agrobacterium-mediated delivery of transgenic constructs as described in ref. ^15^. Transgenic plants were selected on ½ Gamborg B5 with 10 mg l^−1^ hygromycin and 100 μg ml^−1^ cefotaxime.

### EdU labelling of S-phase cells

To visualize S-phase cells in 3-day-old gemmalings, plants were cultured for 3 h in liquid ½ Gamborg B5 medium containing 20 μM EdU followed by fixation in 3.7% formaldehyde in phosphate-buffered saline (PBS, pH 7.4) for 1 h in a vacuum. Plants were then washed twice in PBS and permeabilized for 20 min in 0.5% Triton X-100 in PBS. Next, plants were washed twice in PBS containing 3% bovine serum albumin and placed in the EdU click-IT reaction mixture with the 594 Alexa FLUOR fluorophore in the dark for 1 h (Invitrogen). This was followed by two washes with PBS with 3% bovine serum albumin after which samples were placed in ClearSee solution for 1–4 days, protected from light^16^. Cell walls were stained using calcofluor-white.

### Confocal microscopy and assessment of fusion protein stability

All microscopy was performed on a Leica SP8X-SMD confocal microscope fitted with hybrid detectors and a 40 MHz pulsed white-light laser. Data acquisition was performed using LasX (v3.5.7.23225) software. For mNG, the fluorophore was excited using a 488-nm laser line at 12% power output. Fluorescence was detected between 500 nm and 570 nm with hybrid detectors set to photon counting mode with 1.00–24.50 ns time-gating active to suppress background fluorescence. For the EdU staining experiment, 594 Alexa FLUOR was excited with a 594-nm laser line at 9% laser power and fluorescence emission was captured between 600 nm and 660 nm (0.7–24.50 ns time-gating active). *Z* stacks were acquired using an HC PL APO 20×/0.75 water immersion or APO CS 10×/0.40 dry objective. To display images, ImageJ (v1.52) was used to generate maximum-intensity projections.

When investigating stability of various fusion proteins, we screened T1 transgenic lines for fluorescence by imaging the apical notch region of dormant gemmae. When no fluorescence was detected, we treated these gemmae with Bz (Cayman Chemical) for 24 h or the solvent dimethyl sulfoxide (DMSO; Sigma-Aldrich) as control, and checked for fluorescence again. Transgenic lines that showed no fluorescence before and after Bz treatment were excluded from the analysis.

### Phenotypic analysis

For experiments assessing fluorescence accumulation, non-chimeric transgenic lines were established by growing a G_1_ generation from gemmae of independent plants that appeared resistant to initial selection on hygromycin (T_1_ generation). Gemmae from the G_1_ generation were used for experiments. To assess the phenotypes of plants expressing the full-length MpARF2–mNG fusion, we reported the phenotypes of 50-day-old T_1_-generation plants. For growth assays, dormant gemmae were microdissected from the gemmae cups/thallus, and if clearly distinguishable notches could be observed, these were counted. Next, the gemmae were placed onto ½ Gamborg B5 medium (in the case of 1-naphthaleneacetic acid (NAA) sensitivity, medium was supplemented with DMSO or 3 μM NAA) and grown at 22 °C with 40 μmol m^−2^ s^−1^ of continuous white fluorescent light. After 7 (general growth) or 10 (NAA sensitivity) days, pictures were taken with a Canon EOS250D camera and the thallus area was measured using ImageJ (v1.52). For higher-magnification images, a Leica M205 stereomicroscope was used. Statistical tests were performed in R (v4.2.1).

### Statistics and reproducibility

Tests, parameters, additional statistical information and the nature of biological replicates are provided in the figure descriptions. Sample sizes are shown within the figures themselves. No statistical method was used to predetermine sample size. Sample sizes were determined according to field standards. Experiments were not randomized. For representative micrographs, data collected on two additional independent transgenic lines are provided in the extended data section (Extended Data Fig. 1a–e, Fig. 3c–d and Fig. 7).

### Reporting summary

Further information on research design is available in the Nature Portfolio Reporting Summary linked to this article.

## Supplementary information

Supplementary Information Table containing oligonucleotide sequences used in this study with a short description of their use.

Reporting Summary

## Source data

Source Data Fig. 3 and Extended Data Figs. 4 and 6 Source Data Fig. 3. Unprocessed raw data (thallus area measurements; sheet I) and statistical analysis (sheet II). The file includes an extensive metadata description. Source Data Extended Data Fig. 4. Unprocessed raw data (thallus area measurements; sheet III) and statistical analysis (sheet IV). The file includes an extensive metadata description. Source Data Extended Data Fig. 6. Unprocessed raw data (thallus area measurements; sheet V) and statistical analysis (sheet VI), as well as notch counts of dormant gemmae (sheet VII) and statistical analysis (sheet VIII). The file includes an extensive metadata description.

## Data Availability

Data can be shared upon request. The full phylogenetic tree is available at https://itol.embl.de/tree/13722425211476661730903969. Plasmids generated and used in this study are available upon request from the corresponding author. As the long-term storage of *Marchantia polymorpha* lines is problematic, we can provide some lines analysed in this study (contact the corresponding author for inquiries). Source data are provided with this paper.
