# Supplementary material for: ARF degradation defines a deeply conserved step in auxin response
Source: Nat Plants. 2025 Apr 11;11(4):717–24. doi: 10.1038/s41477-025-01975-1 (PMC12014493; doi:10.1038/s41477-025-01975-1)
Supplement: Supplementary file 1 — Table containing oligonucleotide sequences used in this study with a short description of their use. [file 41477_2025_1975_MOESM1_ESM.pdf]

nature plants

Supplementary information

https://doi.org/10.1038/s41477-025-01975-1

ARF degradation defines a deeply conserved step in auxin response

In the format provided by the authors and unedited

Supplementary Information Table 1

Primer	Sequence 5' > 3'	Description
**MdR298**	TGCATGCCTGCAGGTCGACTAAAAGCCTGTAATCACACGACG	Fw promoter *MpARF2*
**MdR299**	GCTGTCTAGAGGTCGGAACTTCTGTCTAAAATGC	Rv promoter *MpARF2*
**MdR306**	TTAGACAGAAGTTCCGACCTATGTCAGAAGCATCTTCCA	Fw A2 CDS to clone into pA2_XbaI_mNG
**MdR314**	ATGCTGCCGCCGCCAAGCTGCATGTCGTCGCCGCGCG	Rv PB1 to clone into pA2_XbaI_mNG
**MdR308**	CCATGCGGCAACGAAGAAATCTCGATT	Fw E297K mutation and assemble into XbaI site of pA2_XbaI_mNG
**MdR309**	AATCGAGATTTCTTCGTTGCCGCATGG	Rv E297K mutation into XbaI site of pA2_XbaI_mNG
**MdR310**	CGGAGAAATCTCAATTCTCTCTAATTT	Fw R300Q mutation into XbaI site of pA2_XbaI_mNG
**MdR311**	AAATTAGAGAGAATTGAGATTTCTCCG	Rv R300Q mutation into XbaI site of pA2_XbaI_mNG
**MdR312**	ATGCGGCAACGAAGAAATCTCAATTCTCTCT	Fw E297K + R300Q into XbaI site of pA2_XbaI_mNG together with PB1 rev
**MdR313**	AGAGAGAATTGAGATTTCTTCGTTGCCGCAT	Rv E297K + R300Q into XbaI site of pA2_XbaI_mNG together with A2 start fw
**MdR412**	GCGGCAACGGAGAAAAATCGATTCTCTCTAA	Fw S299N mutation into XbaI site of pA2_XbaI_mNG
**MdR413**	TTAGAGAGAATCGATTTTTCTCCGTTGCCGC	Rv S299N mutation into XbaI site of pA2_XbaI_mNG
**MdR315**	ATGCTGCCGCCGCCAAGCTGGAAGGGCTCCACTTCCCATG	Rv A2 AD (DBD end) to clone into pA2_XbaI_mNG
**MdR317**	ATGGCTCCAAAGAAGAAGAGAAAGGTC	Fw A2 DBD to add SV40 NLS at N-terminus
**MdR318**	TTAGACAGAAGTTCCGACCTATGGCTCCAAAGAAGAAG	Fw SV40 NLS to clone into XbaI of plasmid pGWB100 pA2_XbaI_mNG
**MdR319**	GGTTGTAAATTAGAGAGAATTGAGATTTCTCCGTTGCCGC	Rv R300Q mutation into XbaI pA2_XbaI_mNG
**MdR320**	GCGGCAACGGAGAAATCTCAATTCTCTCTAATTTACAACCC	Fw R300Q mutation into XbaI pA2_XbaI_mNG
**MdR372**	TTAGACAGAAGTTCCGACCTATGGCTCCAAAGAAGAAGAGAAAGG	Fw SV40 overhang into xbal with nls
**MdR373**	ATGCTGCCGCCGCCAAGCTGAACTGGACCTTGTTGGAAATTTTGCGACG	Rv MpA2 MR overhang into xbal with nls
**MdR374**	ATGCTGCCGCCGCCAAGCTGCATGTCGTCGCCGCGCGCCC	Rv MpA2 PB1 overhang into xbal with nls
**MdR381**	TGGTTGTCCCTGCACTGCCAGATGGGCAGCGGCCGCGAGAAC	Rv swap degron MpARF3 into MpARF2 DBD longer overhang
**MdR382**	CCCATCTGGCAGTGCAGGGACAACCATTCTCTCTAATTTACAACCCTCG	Fw swap motif MpARF3 in MpARF2 DBD
**MdR424**	GCCCATGCGCTAGCGAGCAGTTCTCCATTCTCTCTAATTTACAACC	Fw swap motif MeARFab in MpARF2 DBD
**MdR425**	TGGAGAACTGCTCGCTAGCGCATGGGCAGCGGCCGCGAGA	Rv swap motif MeARFab in MpARF2 DBD
**MdR426**	CCATGCGGTAAGGCACCTAAAACCATTCTCTCTAATTTACAACCCT	Fw swap motif CsARFab in MpARF2 DBD
**MdR427**	ATGGTTTTAGGTGCCTTACCGCATGGGCAGCGGCCGCGAGAAC	Rv swap motif CsARFab in MpARF2 DBD
**MdR428**	CCATGGGGCAGCGTATGGACAACCATTCTCTCTAATTTACAACCCT	Fw swap motif ScARFab in MpARF2 DBD
**MdR429**	AATGGTTGTCCATACGCTGCCCCATGGGCAGCGGCCGCGAGAAC	Rv swap motif ScARFab in MpARF2 DBD
**MdR430**	ATGCGGCAGCGTCGGGACAGACATTCTCTCTAATTTACAACCCTCGA	Fw swap motif CmARFabc in MpARF2 DBD
**MdR431**	GAATGTCTGTCCCGACGCTGCCGCATGGGCAGCGGCCGCGAGAAC	Rv swap motif CmARFabc in MpARF2 DBD
**MdR359**	GTTCCCGTACTTATCGCATGGGCAGCGGCCGCGAGA	Rv swap motif AtARF2 in MpARF2 DBD
**MdR360**	ATGCGATAAGTACGGGAACTATGTTCTCTCTAATTTACAACCCTCGATC	Fw swap motif AtARF2 in MpARF2 DBD
**MdR389**	CCCATGCGGCAATTTCGGGATCTACATTCTCTCTAATTTACAACCCTCGATC	Fw swap motif SmARF2 in MpARF2 DBD
**MdR390**	AATGTAGATCCCGAAATTGCCGCATGGGCAGCGGCCGCGAGAACCCC	Rv swap motif SmARF2 in MpARF2 DBD
**MdR353**	TTCCCTCCCTTGACGCATGGGCAGCGGCCGCGAGAA	Rv swap motif PpARF2 in MpARF2 DBD
**MdR354**	CCATGCGTCAAGGGAGGGAATGCGATTCTCTCTAATTTACAACCC	Fw swap motif PpARF2 in MpARF2 DBD
**MdR349**	GAATCGAGAATTCGTCGCTGCCGCATGGGCAGCGGCC	Rv swap motif MpARF1 in MpARF2 DBD
**MdR350**	CCGCTGCCCATGCGGCAGCGACGAATTCTCGATTCTCTCTAATTTACAACCC	Fw swap motif MpARF1 in MpARF2 DBD
